# Out of Box Thinking to Tangible Science: A Benchmark History of 3D Bio-Printing in Regenerative Medicine and Tissues Engineering

**DOI:** 10.3390/life13040954

**Published:** 2023-04-05

**Authors:** Karthika Pushparaj, Balamuralikrishnan Balasubramanian, Manikantan Pappuswamy, Vijaya Anand Arumugam, Kaliannan Durairaj, Wen-Chao Liu, Arun Meyyazhagan, Sungkwon Park

**Affiliations:** 1 Department of Zoology, School of Biosciences, Avinashilingam Institute for Home Science and Higher Education for Women, Coimbatore 641 043, Tamil Nadu, India; karthika_zoo@avinuty.ac.in; 2 Department of Food Science and Biotechnology, College of Life Science, Sejong University, Seoul 05006, Republic of Korea; bala.m.k@sejong.ac.kr; 3 Department of Life Science, CHRIST (Deemed to be University), Bengaluru 560 076, Karnataka, India; 4 Department of Human Genetics and Molecular Biology, Bharathiar University, Coimbatore 641 046, Tamil Nadu, India; 5 Department of Infection Biology, School of Medicine, Wonkwang University, lksan 54538, Republic of Korea; 6 Department of Animal Science, College of Coastal Agricultural Sciences, Guangdong Ocean University, Zhanjiang 524088, China

**Keywords:** 3D bioprinting, bioinks, hydrogels, microfluidics, tissue scaffolds, organ-on-chip

## Abstract

Advancements and developments in the 3D bioprinting have been promising and have met the needs of organ transplantation. Current improvements in tissue engineering constructs have enhanced their applications in regenerative medicines and other medical fields. The synergistic effects of 3D bioprinting have brought technologies such as tissue engineering, microfluidics, integrated tissue organ printing, in vivo bioprinted tissue implants, artificial intelligence and machine learning approaches together. These have greatly impacted interventions in medical fields, such as medical implants, multi-organ-on-chip models, prosthetics, drug testing tissue constructs and much more. This technological leap has offered promising personalized solutions for patients with chronic diseases, and neurodegenerative disorders, and who have been in severe accidents. This review discussed the various standing printing methods, such as inkjet, extrusion, laser-assisted, digital light processing, and stereolithographic 3D bioprinter models, adopted for tissue constructs. Additionally, the properties of natural, synthetic, cell-laden, dECM-based, short peptides, nanocomposite and bioactive bioinks are briefly discussed. Sequels of several tissue-laden constructs such as skin, bone and cartilage, liver, kidney, smooth muscles, cardiac and neural tissues are briefly analyzed. Challenges, future perspectives and the impact of microfluidics in resolving the limitations in the field, along with 3D bioprinting, are discussed. Certainly, a technology gap still exists in the scaling up, industrialization and commercialization of this technology for the benefit of stakeholders.

## 1. Introduction

**Figure 1:**
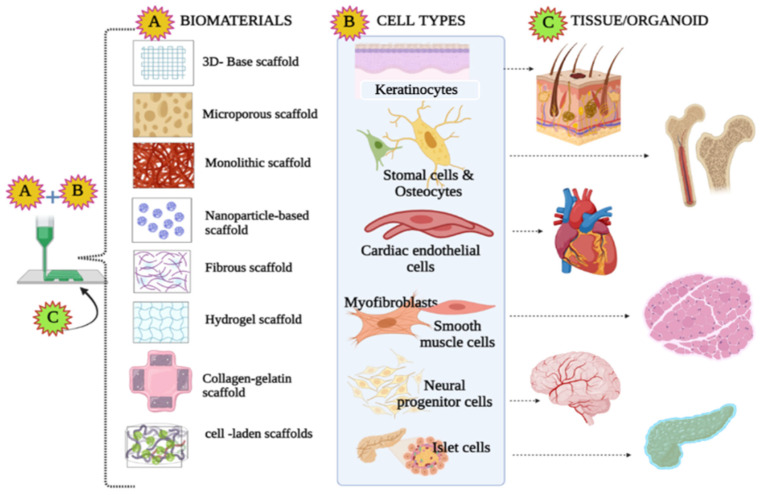
Illustration of different types of biomaterials and tissue constructs used in 3D bioprinting technology.

**Figure 2:**
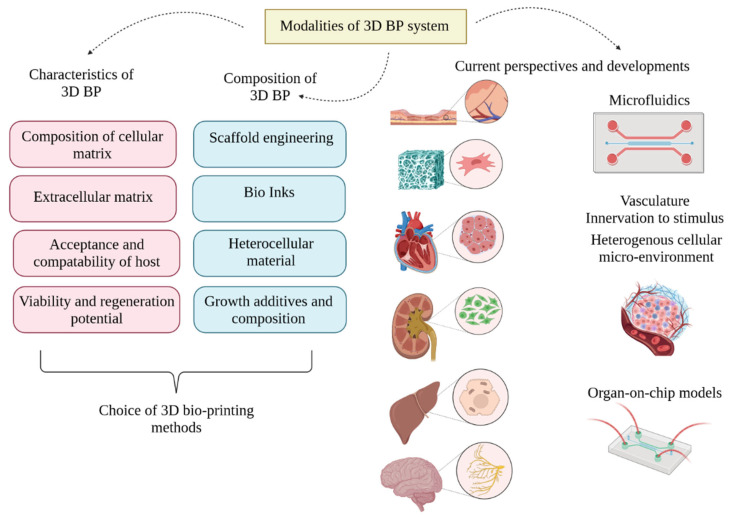
Synergistic effect of microfluidics and 3D bioprinting (3D-BP).

For decades, organ transplantation in the medical field has been high-stakes. The fundamental challenge is the increasing demand for organs and decreasing number of organ donors. However, the donation process is complicated and involves several protocols such as opt-in, opt-out policies, types of donation (e.g. deceased and live donors). consent mechanisms, etc. Consequently, medical researchers have constantly looked up to offer solutions to boost the organ supply [1]. This debate is long-lived, and over the last few decades, there have been progressive developments in the parallel tissue engineering domain involving 3D printing. Certainly, 3D printing and 3D bioprinting (3D-BP) are different; the adoption of bioinks for mimicking tissue and organ systems was a pressing need in order to manage organ transplantation-related requirements. The combination of fabrication and clinical practice has been the fundamental basis for the present-day 3D-BP and tissue engineering. The major task ahead in the field is to determine the tangible scalability and mechanical properties of the biomaterials to match the anatomical features, as in most cases they fail to fit the injured site. Additionally, the self-repairing mechanism is a major lacuna, and its prominent functionality should be studied in order to enhance the connectivity between the tissues and the musculoskeletal system. Further, minor damage to the implanted biomaterial may be capable of self-healing, and interference with the surrounding tissues could extend the damage. Therefore, more intricate knowledge on developing biomaterials in vivo should be developed. Inclusion of and emphasis on the ample medical findings on tissue-specific interaction should be explored, and integration of ideas such as mimics should be implemented to overcome the shortcomings of the domain. In a nutshell, tissue engineering is technology that imitates the interplay between cells and their habitats or surroundings; however, a response in phenomena from the engineered cells to the extra and intracellular signals remains a challenging task in this decade. Regenerative medicine is evolving rapidly with the aim of replacing and repairing the functional properties of damaged tissues and organs. The process of 3D printing involves the production of mimics of natural tissues using bioinks and other biomaterials such as living cells. Figure 1 illustrates the different bioinks, scaffolds and biomaterials used in 3D-BP. The human body is not as complicated as it looks; it is designated an organ-grade organization formed of homologous tissues and cells that perform similar functions. It is self-assembled basically by four major tissue types, nervous, epithelial, muscular and connective tissues, each with specific histological characters. Historically, 3D techniques have opted to focus on histological intricacy, using various biomaterials and technologies to tailor functional organs [2]. The fatal stage of organ failure requires functionally active organs and tissues which differ in their morphology and physiology; however, the bioengineered material must be intricately identical. For engineering a bio-transplantable organ, several governing factors such as choice of scaffold, establishing vasculature, innervations and regeneration potency, selection of bioinks, knowledge on immunological responses, the physiology and working of the transplanted organ, and a durability and life-span assessment must be studied very cautiously [3]. Apart from these, decellularization of organs using 3D-BP as a scaffold for developing new organ transplants through a recellularization process had showed promising results [4]. Further, this technology proposed the assemblage of bioinks using the donor’s cells, which led to substantial decrease in immunological rejection and improved the success rate in fabricated in vitro models and surgical implants. A critical evaluation was carried out among the eight organ systems including skeletal, endocrine and exocrine, gastrointestinal, respiratory, cardiovascular, urinary and integumentary systems [5]. 3D-BP is a groundbreaking technology that enabled the mimetic assemblage of the complex tissue microenvironment and provided mechanical support and intricate detailing of the cytoskeleton through bioink fabrication techniques. These developments offer avenues for solving demand for organ donors and organ transplantation. Additionally, 3D-BP provides sustainable advancement in regenerative medicine through bio-printed transplantable tissues relating to the eleven organ systems of the human anatomy. Further, 3D-BP technology was influential in contributing to advancements in in vitro drug testing models, drug developments and delivery systems. However, the scalability of 3D-BP was significantly improved through computer-simulated models. The critical challenge of these was based on the sustenance of post-printed blended tissues and the stability of bioinks. Further, stimulatory studies aided in the testing and prediction of different bioprinting modalities, the development of novel bioinks with customizable properties, and understanding of their interactions within the cell environment. In future, AI and IoT-based 4D BP would aid in addressing future requirements in the clinical industry. 3D-BP technology is also used in engineering medical and surgical devices, and clinical station models for experimental learning and surgical implants [6]. Therefore, the ultimate goal is to attain uncompromised scientific and clinical achievement to redefine the medical field and to address the ethics, moral values and opt cues of restoring life. One of the prime challenges in 3D-BP is developing intra-organ vascular structures composed of arteries, veins and capillaries. These are crucial for supplying blood and oxygen to the tissues that are necessary for the viability of tissue constructs. Specific tissues such as cardiac and liver tissues require ample oxygen consumption for an adequate supply of nutrients to prevent necrosis. Existing fabricated blood capillaries have a resolution of up to approximately 20 μm; however, the required blood capillary diameter is as small as 3 μm. Bioinks with angiogenic growth factors have been employed to induce vascularized structures post-bioprinting [7]. On the other hand, microfluidics has been applied to create vasculogenesis; apparently, the hydrogels fail to produce sustenance in cellular interactions and negatively impact the stability of the tissue constructs [8]. Hence, this is also considered to be a research gap involving microfluidics in the development of functional vasculature in 3D-BP models. The present review discusses the applications of 3D-BP as an effective tool in translational and regenerative medicine, and considers the challenges of developing 3D-BP tissue types such as skin, bone, cardiovascular, kidney and liver. Moreover, the development of 3D scaffolds similar to natural tissues through different printing methods is also discussed. With emphasis on regenerative medicine, the adoption of natural peptides and synthetic polymer-based bioinks is also highlighted. Further, the synergistic effect of microfluidics and 3D-BP adds significance to and advances the technique (Figure 2).

## 2. Sequel of Skin Tissue Models

Due to the simple structure and design, skin was one of the earliest studied models in 3D-BP; however, it has been reported that non-3D-BP models existed in the mid-1990s. The focal aim of tailoring skin models was to offer effective alternatives for skin grafting, wound healing and other chronic skin-related diseases. Nevertheless, cosmetic skin medicaments and surgeries have now been increasing in such a way that demands 3D-BP skin models. Several non-3D-BP models include artificial skin-like synthetic silicone layers which were used as commercial skin substitutes, such as Biobrane and Integra [9]. There are also natural human acellular tissue substitutes derived from human cadaver dermal layers and further cultured under controlled in vitro conditions (e.g., Alloderm, Graftjacket). With minor tissue culture interventions, another kind of commercial non-printed 3D-BP skin model used allogenic-human foreskin fibroblast cultured on a synthetic polyglactin scaffold with metabolically active secretions (e.g., Demograft). Similarly, Apligraft was developed through in vitro culturing of neonatal fibroblasts from human foreskin on dual matrix layers closely mimicking the dermis (bovine type I collagen) and epidermis (epidermal keratinocytes) [10]. The aforementioned are the clinical success stories; however, these models did not replicate the composition of natural skin. More precisely they lack the functionality of the skin due to their lack of blood vasculature, stimulation and innervation, and their absence of sebum, pigments and hair follicles. In other words, the major drawbacks included extended fabrication time, non-tailored and un-personalized approaches to meet the unique demands of patients, and infections in host cells and skeletal and musculature systems [11]. 3D-BP of skin tissue was designed to address the lacunae observed in non-bioprinted skin models. The pipeline involves the choice of specific skin type cell lines, biomaterials, in vitro culture systems, and safety and evaluation testing. For developing 3D-BP skin models, specific cell lines such as HEKn, HEKa, HaCaT, K38 (keratinocytes), HDFa, HDFn, NIH3T3 (fibroblasts) and HEMa, HEMn (melanocytes) are the mandatory requirements. 

In addition, other types of cells may be chosen depending on their requirements; these include glandular cells, stem and progenitor cells, nerve cells, etc. Natural and synthetic biomaterials such as peptides, collagen, polyethylene glycol (PEG) and sodium alginate have thus far been the best choice. Traditional (laser-induced, multi-material and extrusion) [12] and recent bioprinting techniques (electrohydrodynamic, microfluidic and supramolecular polypeptide-DNA hydrogels bioprinting) have been adopted [13]. Further steps involve cell differentiation, progression, and maturation, followed by biochemical, immuohistocompatability and mechanical testing. In recent trends, research efforts in 3D-BP have played a pivotal role in developing artificial skin models in the cosmetic industry, and have paved the way in providing cues for preventing graft rejection, with radical functionalities being developed. Several works have been established, incorporating melanocytes using tyrosinase-doped bioinks that have been produced to produce a 3D-BP skin [14]. Customized techniques such as control of the rate/distribution/location of melanocyte deposition have successfully been incorporated in 3D-BP skin models to achieve a close resemblance to the native skin [15]; there are also freckle-free skin models [16] and infusion models with melanocytes differentiated from the pluripotent stem cell (iPSC), facilitating a greater transformation of melanin to keratinocytes [17]. Improvisation of vascularization was successful in producing vascularized skin patches using a combination of endothelial and adipose-derived stem cells [18], and integration of de novo vasculogenesis through encapsulation of human umbilical smooth muscle cells’ sodium alginate-collagen matrix demonstrated the feasibility of vasculature in 3D-BP skin models [19]. Innervation was stated as one of the drawbacks of non-BP skin models, and this is more significant when it comes to accurate understanding of the allergic, pain and drug responses of the grafted skin, both in patients and also in in vitro representation. Impregnation of nerve cells in the dermal region was trialed in the burned skin of mice by embedding the skin matrix with keratinocytes in a collagen hydrogel [20], while in a similar study, the sensory neurons were implanted in an artificially tailored tissue, and it was observed that the nerve cells progressed and penetrated into the epidermal layer after the incubation period [21]. In another study, 3D-BP models were used for the reinforcement of laminin-filled micro channels for managing the growth of the neurons [5]. However, more experimental studies for innervation are needed to explore fully functional 3D-BP skin models.

## 3. Sequel of Bones and Cartilaginous Tissue Models

Unlike other tissues, bones impart hardness and mechanical support to the skeleto-muscular system. On the other hand, cartilages are the soft elastic flexible connective tissues which function as the bridging/connecting link that bears the pressure and loads; they are important for seamless fluid–joint movements. Therefore, it is a highly challenging task to advance research on 3D-BP to offer solutions for bone and cartilage injuries. Several capable 3D-BP clinical translation and in vivo models are experimented with in this platform to offer viable alternatives for bone injuries. Recreating the bone architecture through scaffolds and simulating the in vivo cellular environment was the prime objective in 3D-BP bone models. Fabrication on amorphous calcium phosphate (ACP)/calcium sulfate hemihydrate (CSH) composite scaffolds using alginate/cellulose gels with varying concentrations of 0–23% positively impacted the structural, physiological and biological properties of bone tissue models [22]. Strengthening of bone constructs using bioceramics with phosphate powders obtained from trimeta- and tripolyphosphate inhibitors was reported [23]. Amorphous calcium phosphate (ACP) is the known to be the precursor for hydroxyapatite (HAP), which is largely considered significant in bone tissue engineering [24]. In this context, nanodispersed ACP is used synergistically with calcium phosphate powder in ceramic implants due to its particle size, up to sub-microstructures [25]. The choice of progenitor cell sources, such as human osteoblast cell lines, is limited; however, human mesenchymal stem cells (MSCs) exhibit potency for bone regeneration. In the context of the above, experimental studies in vivo using rabbit models have been validated with a combination of MSCs with collagen–hydroxyapatite scaffolds in bone repair [26]. Intervention with nanotechnology in bioinks for 3D-BP bone models for better adhesion and bone cell differentiation was demonstrated using hydrogels composed of polylactic acid (PLA) and arginylglycylaspartic acid (RGD)-conjugated nanoparticles [27]. Improvisation on the adhesiveness and augmentation of mechanical support was validated using a combination of agarose and collagen hydrogels, which in turn impacted osteogenic differentiation of human MSCs [28]. The selection of biomaterials for bioinks governs the histological environment of the target tissues and ensures appropriate design facets for 3D-BP of bones and cartilages. Several studies have utilized different synthetic biomaterials such as polyethylene glycol (PEG), dimethacrylate (PEGDMA) [29], alginate-nanocellulose, HA and gelatin methacrylate (GelMA) [30]. The differentiation of MSCs was dependent upon biomaterials such as agarose, alginate, GelMA and PEGDMA [31], and was experimentally proved in a study which involved encapsulated MSCs laid on RGD-functionalized PEGDMA hydrogel [32]. Very recently, a silk-based natural polymer, silk fibroin methacrylate (SilMA), exhibited an appreciable potency in improving cell adhesion and fabrication of scaffolds in bones and also in cartilage. In a nutshell, the development of 3D-BP of bone models relies on the selection of biomaterials that reciprocate their efficiency in terms of mechanical support, tensile strength and mimicking the in vivo microenvironment. The cellular interaction of 3D-BP is one of the most challenging tasks; however, vascularization of 3D-BP constructs was made possible through multi-nozzle printing using MSCs with human umbilical vein endothelial cells (HUVECs) and human neonatal dermal fibroblasts (HNDFs) [33]. The HNDFs critically improved permeability, which facilitated diffusion and served as a functional binding layer in the 3D impregnated microenvironment. Validation of cartilage regeneration in in vivo models was proven, wherein the human chondrocytes and human MSCs demonstrated proliferation and growth up to 17%, and secreted type 2 collagen and glycosaminoglycan on the implanted surface after 62 days of incubation [34]. Clinical trials are underway using 3D-non printed models; however, a partially completed study on articular cartilage regeneration using human MSCs undertaken by Tehran University of Medical Sciences (ID: NCT00850187) was reported. Recent studies have also shown the impact of physiological activity levels and immersion duration on the durability and life of porous magnesium scaffolds. Loss of relative surface area is evidenced through dynamic immersion testing and micro-tomographic images which provide insight to monitor the fatigue of the implants and the bone healing process [35]. As a conclusive point of view, bones and cartilages are tissues with simple anatomy and minimal vasculature compared to other organs; in spite of much research, translation from laboratory bench to clinic is a challenging and demanding task. As mentioned earlier, culture media, biomaterials and nutrient-enhancers are derived from synthetic sources or animal-derived substances; thus, there is a greater probability of developing immune responses and hyperallergic reactions after implantation in the host. Generally, stem cells are used as progenitors, which in the long term may cause teratomas (stem cell cancer), which can be disastrous [36]. Ample experimental and clinical validation is in progress; however, solutions to the aforementioned challenges should be found in order to make this technology seamlessly available to stakeholders.

## 4. Sequel of 3D-BP Cardiac Tissue Models

Cardio-vascular diseases and valvular deformities are common in the neonates to older cohorts in the current population, who usually undertake replacement surgery with a range of options categorized based on cost-effectiveness and the nature of the material used (synthetic, mechanical or bio-based prosthetic). The efficiency of the implanted valve diminishes along with time, and this warrants more surgeries for efficient functionality and durability [37]. Hence, there is an increased requirement for biologically derived valves that could remain compatible for life. 3D-BP offers a platform for bioprinting valves using natural biomaterials such as alginate/gelatin [38] and methacrylated gelatin [39], with aortic valve interstitial cells and smooth muscle cells (SMCs) which mimic the histological and mechanical properties of the cardiac valves [40]. 3D-BP offers yet more solutions for cardiac deformities; these include cardiac patches which are engineered using cardiomyocytes isolated from neonatal rats and complex fibrin-based materials, and are laid on a hydrogel matrix that mimics the innate myocardium [41]. Another significant leap in the development of cardiac patches was the use of a combination of alginate and PEG-fibrinogen with HUVECs and iPSC-derived cardiomyocytes, which demonstrated effective amalgamation with the host tissues and regenerated vasculature [42]. Construction of cardiac spheroids was a bench-mark in the evolution of cardiac patches, which successfully produced vascularization in the in vivo models [43]. It is important to mention the complexity of cardiac tissues in terms of mechanical flexibility, contractions, vascularization, innervation and electrical stimulation; further, more intense research is needed to fill the above lacunas in order to enter the next phase of clinical trials. With sustained development, 3D-BP cardiac tissues and patches will undoubtedly offer tangible solutions to patients with heart-related diseases in the forthcoming years.

## 5. Sequel of 3D-BP Kidney and Liver Tissue Models

Within the growing population, metabolic disorders occupy a substantial percentage; kidney failure and renal-associated dysfunctions are very common. Renal disorders are likely to be associated with chronic diseases such as renal cancer and diabetes; however, prolonged intake of drugs for any ailment leads to a greater possibly of acquiring renal failure in due course. The suggested medicaments for treating renal problems vary according to severity; however, periodical dialysis, kidney transplantation, and bio-artificial kidneys are a few of the viable options [44]. Prominent drawbacks associated with these options include non-availability of a donor, graft rejection, heightened immune hypersensitive reactions, failure to substitute the overall renal metabolism, or a less efficient metabolism. Further, in drug testing and development, nephrotoxicity assessments principally rely on the renal functions; thus, there is a major requirement for in vivo renal models [45]. In order to satisfy rising demands, 3D-BP has driven steadfast approaches to developing bioprinted renal tissues. The nephron is the functional unit of the excretory system in humans, and a greater part of absorption and re-absorption takes place in the proximal convoluted tubule (PTC) of the nephron [46]. Therefore, much importance is placed on the development of 3D-BP PTC to recreate its function in in vitro renal models. 3D-BP models of human PCT were devised and embedded in the extracellular matrix on a modified perfusion scaffold for the formation of epithelial layers (EL) [47]; this entire model mimicked the structural and functional properties of the renal tissue. However, the EL were dislocated on exposure to nephrotoxic drugs at increasing concentrations. This was considered a drawback of the in vitro 3D-BP models. A complete human in vitro PCT model mimicking the histology and physiology of renal tissues, which constituted the layers of endothelial cells and fibroblasts tailored to strengthen the EL, was developed through the Organovo 3D-BP platform [48]. This model was an advancement; it rectified the lacunae posed in the earlier studies and was resistant to the drug-induced nephrotoxicity (cisplatin). Bioprinting of organs is challenging because these organs are connected with systemic circulation and are highly vascularized. They are built-up with more than twenty-five types of cells, with precise networks and histological properties. As of today, a whole bioprinted kidney model is not available; however, due to constant improvisations and technological advances, it may soon be available to reduce the burden on renal disorder patients. Human iPSC-derived hematopoietic progenitor cells (iPSC-HPCs) have been the prime choice for 3D-BP liver tissue culture in vitro. It is a well-known fact that the liver is the largest gland, performing vast metabolic and physiological function. Mimicking its intracellular compartments and intricate architecture is undoubtedly a challenging process. A tri-layered 3D-BP model comprising iPSC-HPCs, HUVECs, and adipose-derived stem cells mimicking the natural liver was developed and physiologically functional [49]. Similar to cardiac patches, a study reported the utilization of a liver decellularized ECM scaffold 3D-BP model in the cirrhotic liver, which effectively substituted the defective tissues and improved the performance [50]. The role of the 3D-BP liver model in regenerative medicine is to understand the pathophysiological effects after drug administration and constructively use them to replace the diseases and damaged liver tissues in patients. The human iPSC-HPCs demonstrated similar functions to those of hepatocytes, and served as the principal choice for biomaterials with an alginate hydrogel scaffold [51]; however, HepG2 cells were also experimented with the alginate matrix [52]. In advanced 3D-BP liver models, the internal micro-architecture was strengthened by a polycaprolactone-laid capillary framework which imparted synergistic effects for the cell proliferation. The combination of the three types of cell culture, hepatocytes, HUVECs, and human lung fibroblasts, imparted standard levels of albumin and urea production, which amplified the survival rates and spontaneous cellular reorganization [53]. The liver as an organ has a self-regenerative capacity; therefore, tailoring of the entire organ is not required, but producing 3D-BP liver tissues to substitute the repaired tissues may be effective.

## 6. Sequel of Neural Tissue

One of the biggest challenges facing both the healthcare and medical system is finding solutions for the replacement of severed neurons and developing nervous tissues in in vitro culture conditions. A critical component of successful nerve regeneration is 3D scaffolds, which provide essential mechanical support to expedite the cellular functions that result in improved host–tissue engraftment and consequent fresh tissue development. The domain of neural tissue engineering is greatly evolving due to the development of 3D-BP, which utilizes biocompatible hydrogels, bioinks and scaffolds for better cellular responses [54]. 3D-BP constructs have enabled research into cellular and cell–extracellular matrix communications in a species-specific, high-throughput manner, which has facilitated the screening of drugs and targeted delivery platforms. The choice of bioink for 3D-BP of the neural cells/tissues is very limited. Artificial neural tissues were constructed using bioinks developed from vascular endothelial growth factor (VEGF), murine NSCs, collagen, fibrin gel [55] and retinal glia cells [56]. In a similar study, PBMCs-derived iPSCs and iPSCs-derived NSCs showed better viability and cellular communication required for further mechanistic studies [57]. Current 3D-BP platforms have provided solutions for glioblastoma (GBM), which is a severely immunosuppressive state with a complicated and assorted tumor microenvironment. The prognosis is much limited by constrained drug delivery through blood–brain barrier (BBB) systems. To overcome this hurdle, current 3D-BP GBM and BBB models have provided intricate biomaterials to mimic and fabricate the natural native tissues. They provide alternatives for reliable physiological and mechanistic studies featuring drug screenings and also accelerate drug development [58]. Remarkable advancements have been made across the choice of biomaterials used in 3D-BP nerve conduits; in this context, live Schwann cells encapsulated in scaffolds comprising composite hydrogels of alginate, fibrin, hyaluronic acid, and/or RGD peptide were engineered. Several physical and mechanical parameters were investigated in parallel with the enactment of Schwann cells within the bioprinted scaffolds. The results showed that a better performance of the Schwann cells was recorded based on the viability, propagation, coordination, placement and ability to produce laminin. These results demonstrated haptotactic signals through the extension of dorsal root ganglion neurites along the bioprinted strands, thus demonstrating prospects for applications in the field of nerve tissue engineering [59]. Clinical investigations on the repair of spinal cord injury (SCI) in vivo using 3D-BP constructs composed of neural stem cells (NSCs) were studied. A novel composite bioink made of functional chitosan, hyaluronic acid derivatives and matrigel demonstrated rapid gelation and cross-linking potency, which promoted one-step bioprinting of spinal cord-like constructs. Specific challenges such as technical face-offs in the printing process, deprived cell viability, and negligible cellular interactions in the 3D-BP spinal cord-like constructs were reduced in the study. The SCI model rats showed substantial locomotor recovery as the 3D-BP scaffolds stimulated axon regeneration and decreased glial scar deposition [60]. Adult zebrafish models with induced CNS deficits and traumatic brain injury implanted with the 3D-BP NSC-laden PU constructs showed significant locomotor function recovery, with a reduced mortality rate that provided in vivo clinical evidence to support the efficacy of 3D-printed NSC-laden constructs in repairing CNS diseases [61]. Table 1 represents the clinical evidence of 3D-BP models in different types of tissues.

## 7. Bioprinters and Bioinks for 3D Printing

**Figure 3:**
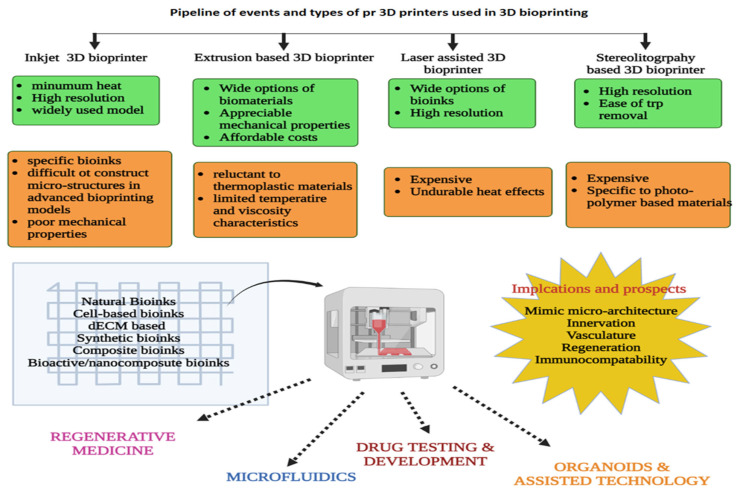
Illustration of the types of bioinks, pros and cons of 3D bioprinters and applications of 3D-BP.

The constituent’s elements for the 3D printing technology involved high temperature setup and harsh chemicals which were seeded to develop non-biological applications. However, this composition was not appropriate for creating the biological materials involved in 3D-BP; thus, it is crucial to fit appropriate biomaterials that match the structural, functional and mechanical properties of the natural tissue. In simple terms, bioink is a mixture of several biomaterials (cell lines of choice) in the form of hydrogel which are cross-linked immediately after bio-printing to achieve a desired shape, structure and micro-architecture of the targeted organ or tissue [72]. There has been a lot of exploration on bioprinting methods; they are generally classified as laser-assisted bioprinting (LaBP), inkjet bioprinting/droplet bioprinting (IJKBP), and extrusion-based bioprinting [73], fused deposition modeling (FDM), direct ink writing (DIW), selective laser sintering (SLS), stereolithography (SLA) and laser-induced forward transfer (LIFT). However, DIW and IJKBP are commonly chosen for 3D printing of live cells [74]. Figure 3 depicts the pipeline of events in 3D-BP. Due to rising demand, new modalities have been put to use; one such model is a multi-head deposition system (MHDSs), which operates on a CAD-programmed platform for designing constructs in a more precise manner [75]. 

The endurance to printability is a significant property of an ideal bioink model, wherein it should possess the texture to fit in prescribe in the scaffolds and provide the anticipated structure and 3D-resolution. Further, the physio-chemical characteristics should work in such a way as to augment the cell proliferation, flow and uniform fabrication and improve the cell viability [76] post-printing, thereby making the 3D-BP model sustainable. More specifically, in regenerative medicine, biocompatibility becomes a prime factor of the chosen bioinks, in order to mimic natural environment for the cell to proliferate and be sustained. Most importantly, they should provide support for adherence in terms of physical and mechanical sustenance that maintains the 3D cellular environment [77]. Significantly, the bioink should be able to provide a seamless microenvironment and offer support during the degeneration of the constructs, and should simultaneously uphold the regenerative potency of new tissues [78]. The bioinks were initially categorized as natural polymers, synthetic polymers an poly (ε-caprolactone) (PCL); however, due to multi- and interdisciplinary advents, bioinks with novel properties such as ultrashort peptides are used [79]. HDG-BI are generally categorized into protein-based, polysaccharides, dECM-based bioinks, and synthetic polymer-based bioinks. While the constituents of hydrogel-based bioinks (HDG-BI) include sodium alginate (alginate), collagen and gelatin, hyaluronic acid, silk fibroin, fibrin/fibrinogen, gellan gum, agarose, chitosan, silk, decellularized extracellular matrix (decm), poly (ethylene glycol) (peg), and pluronic [80]. Some commercial bioinks have recently been launched, including Dermamatrix, NovoGeI [81] and cellInk [82], which offer more advantages and efficiency in performance. Another type of bioink is the cell aggregate/pellet-based bioinks which are chosen for direct application of cell aggregates in scaffold-free tissue fabrication. The most advanced technologies are composite bioinks and bioinks with bioactive molecules, wherein the nanomaterials are employed to work hand-in-hand with the hydrogel biomaterials. In the latter case, growth enhancers such as proteins, hormones and other stimulating factors responsible for growth and differentiation are added up in the formulation of the bioink [83]. Table 2 illustrates the various types of bioinks that are currently used for tissue constructs. 

## 8. 3D-BP and Micro Fluidics

**Figure 4:**
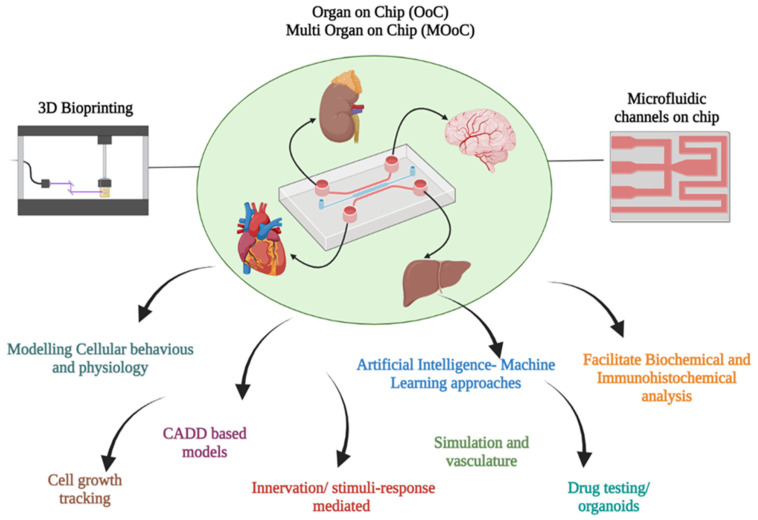
Combined effects of 3D-BP and microfluidics in development of organ-on-chip models.

3D-BP techniques integrated with micro fluidics (MF) offer solutions for the limitations such as construction of micro-tissue architects equipped with vascularization and innervations. The precise choices of nozzles facilitate the placement of bioinks in the intricate tunnels with maximum accuracy, and therefore the achievement of printable quality. Utilization of MF channels and chambers enable 3D printing on a chip which can optimize the innervations or response to stimuli from external environments. Thus, this facet of complex built-in micro channel structures helps in the organization and coordinated functioning of in vivo network-like structures such as blood vessels, which improve the vasculature of the 3D-BP tissue and organs [113]. MF-assisted 3D-BPs are advantageous over conventional 3D constructs in several ways, one of which is their construction of detailed and intricate structures that closely resemble real ones, through augmentation of proportional volume and dimensions. Secondly, recovery of ensuing samples such as proteins/growth factors for testing is effortless, which helps in validating the mechanisms in the printed tissue. Lastly, they have the unique feature of controlling and monitoring the mechanism of the MF-3D-BP construct through an external automated peripheral device [114]. Proof of this concept has been demonstrated through experimental studies which presented the lung-on-a-chip model, thereby attracting scientific cohorts. Meanwhile, an MF skin-on-chip system was constructed using hydrogels consisting of primary human skin cells and ECM in a double-layered polydimethylsiloxane (PDMS) platform. The basement was designed with intricate micro channels to simulate the vasculature and physiological transport systems in an organ-on-chip (OoC) model [115]. Similarly, bone-on-chip MF-3D-BP models were engineered with a bone matrix consisting of mineral and collagen, which contributed to the building of fundamental properties of the bone architecture. Furthermore, the re-created bone model offered spatial resolution and patterns for filling the immobilized biomatrix embedded with growth factors for regeneration [116]. Brain injury can be a direct physical trauma caused by an accident or a chemically inflicted trauma caused by toxicity, inflammation by free radicals, neuro-degenerative disorders, ischemic shock or the side-effects of head injury [117]. Research and clinical efforts to use 3D-BP nerve models have come across several limitations, such as bulky tissues, lack of innervations and vasculature and uncontrolled microenvironments [118]. The MF OoC models have been developed to study axon and glial cell growth, intra- and extracellular signaling, synaptogenesis and other neurovascular functions. Specifically, brain-on-a-chip (BoC) models have addressed the existing drawbacks by incorporating micrometer-scale cell layers with compartmentalized neural chambers filled with immobile or dynamic fluid conditions; all these advanced developments facilitate ample access for metabolite exchange, and maintain the variability and micro physiological function of the BoC models [119]. Recently, a BoC-OoC model was fabricated through soft lithography 3D-BP using polydimethylsiloxane (PDMS) and neural organoids-spheroids. The model was articulated by micro-pillars and embedded with copper electrode sensors for the detection of electro-chemical stimuli [120]. The drug development process (DDP) involves pre-and post-clinical phases which involve testing in in vitro, in vivo and in human trials. The OoC models facilitate the DDP through facilitating drug testing in the OoC models, which are bound to 3Rs principles (the Replacement, Reduction, and Refinement Directive, 2010/63/EU). Drug testing principally involves the liver, kidney and heart; thus, it is of paramount importance to devise multi-organ chips (MoC) that can dramatically contribute to lessening the costs, duration and ethical laws enforced on in vivo studies. Further, the micro physiological design restores the functional structure of the organs with natural functions and features that can benefit the drug trials [121]. A MoC model composed of six organoid systems, viz., liver, heart, lung, vascular, testis, brain or colon was devised by Skardal et al. [108] for dose-dependent drug studies. The MoC comprised multiple layers, viz., a lid chamber layer, a PMMA sheet, a microfluidics layer, and a porous membrane layer. The biomaterials of liver organoids are composed of PHHs (80%), HSCs (10%), KCs (10%), whereas cardiac organoids are composed of 90% of iPSC-CMs and 10% human primary cardiac fibroblasts. The liver and cardiac organoids were tested with a series of drugs for hepatoxicity and cardiotoxicity; however, several responses were mimicked by the response in the MoC microfluidic system [108]. In another study, the microfluidic 3D-BP approach was employed to develop urethral tissue scaffolds using a PLGA/PCL/TEC polymer loaded with L929 cells, as biomaterials, along with with GelMA, alginate, and PEGA derivative bioink; this study highlighted the growth of cannular tissue, which may be used in tissue regeneration [5]. Thus, the 3D-BP and MF are co-evolving multi-disciplinary fields benefitting the regenerative and clinical medical domains. Figure 4 demonstrates the interdisciplinary impacts of creating OoC and MOoC models integrated with 3D-BP technology. Advancement can be observed in the successful demonstration of experimental in vivo and in vitro models, pre-clinical implants, OoC and MoC; however, more of proof of concepts of translation of bio-printed tissues/organ equivalents is needed for scalability.

## 9. Significance of 3D-BP in Tissue Regeneration

**Figure 5:**
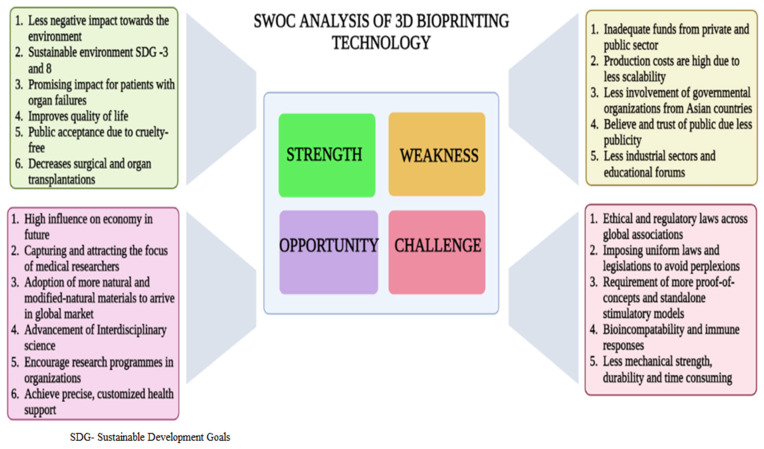
Representation of the pros and cons of 3D-BP through SWOC analysis.

Tissue engineering and 3D-BP have synergistically impacted on the field of regenerative medicine in the past two decades to offer solutions for organ disorders and related ailments. The application of hydrogel-based cell inks has prompted progress in making successful 3D-BP tissue constructs. They are customized as per the patient’s specific needs, and exhibit high levels of biocompatibility and regenerative ability, increased viability, and deceased rates of organ rejection; all these factors may possibility offset the major drawbacks of organ transplantation. Nevertheless, several other factors such as manufacturing cost, the mechanical strength of biomaterials, and the durability and essentiality of multi-cellular printing are limitations. Vasculature is also one of the deficit features in many 3D-BP models; further advancements are required to develop functional vascularized structures in 3D-BP. It is anticipated that the future of 3D-BP is 4D bioprinting, wherein the bioprinted structures possess the feature of re-modelling their shapes and structure in response to an external stimulus [122]. Development of on-site, handy 3D-BP for use in clinics, with which the physician can instantly apply the 3D-BP (e.g., skin patches) with less pain and exposure time, is also a welcome idea. Shear stress and pressure during extrusion printing greatly impacts the cell viability in 3D-BP constructs; however, progressive technologies such as fluid-dynamic models have curtailed this stress through optimization of nozzle dimensions and the viscosity of bioink (e.g., a blend of alginate and gelatin methacroyl (GelMA)), thereby improving cell viability [33]. Yet another benchmark in 3D-BP is the glycemic correction in insulin-dependent patients through bio-printing of extra-hepatic transplantation of islet cell constructs. This hydrogel construct mimics the native cells and has demonstrated permeability of oxygen and nutrients to maintain viability; thus, it offers a solution for islet transplantation in diabetic patients [123]. Yet another milestone is the intervention of 3D-BP in in vivo organ sites, at which the new 3D-BP tissue constructs are directly placed at the defective organ’s sites through probes during surgical procedures; the validation of this technique was experimentally proved through replacement of the osteochondral bone in mice, using n-hydroxyapatite hydrogels in the defective site [124]. Figure 5 demonstrates the SWOC components in 3D-BP techniques.

## 10. Limitations

3D in vitro structures have progressed the drug screening process, while 3D tissue models can meticulously mimic the physiological responses of native tissues. 3D-BP is considered a highly promising technology amongst other in vitro systems, with the advantages of custom-made microarchitecture, high-throughput ability, co-culture capability, and reduced risk of cross-contamination [125]. Moreover, the huge market demand for functional skin substitutes in the cosmaceutics industry has had great impact after the ban imposed by the EU on cosmetic testing on animals [126]. Meanwhile, current 3D-BP skin models require improvement in providing necessary functional units, which certainly hinders their advancement. Further limitations of 3D-BP models may be the constraints of involving different cell types and the large numbers of cells that are tailored into complex structures [127]. ECM-derived matrices exhibit variability in their biological features due to modified and varied production levels [128]. However, a few studies have reported that certain ECM constituents and natural scaffolds were not ideal and consistent for drug testing and other toxicological assays [129]. Other challenges include a lack of vascularization networks, limitation of drug diffusion to the target core, and expensive outlay for large-scale production. However, these shortcomings apply to low-grade biomimetic cell inks with heterogeneous micro-architectures and low stability. Contrastingly, fabricating intricate micro-architectures with precise bioinks and ECM components can restore the ideal functionality of organs. Generally, bioprinting approaches involve a homogenetic or similar tissue construction; however, developing this is essential to exactly mimic organs which usually constitute a hetero cellular anatomy and architectures [130]. Another prominent challenge is developing scaffold designs to satisfy biological requirements such as cell seeding, differentiation, proliferation, vascularization and innervation. Tailored scaffolds should have insights into porosity, surface tension, curvature, inter-connectivity, tortuosity, pore shape, size and gradation; these are to be taken as crucial parameters for mimicking the histomorphometric architecture of native cells [131].

## 11. Tangible Outcomes and Future Outlook of 3D-BP

The best utilization of 3D-BP models can be achieved by integrating microfluidics and by development of tissue/organ-on-a-chip models through high-throughput screening and 3D array platforms. They provide avenues for better cell–cell signaling, perfusion and target delivery of drugs, shear stress and regulating biochemical stimulation in the in vitro environment. The lack of vasculature in 3D-BP models was the prominent drawback; however, this can be remedied through the utilization of fugitive ink for perfusable channel fabrication, or through biologically sprouting capillaries and anastomosing them between organoids. A real-time prototype of a perfusable pancreas-on-a-chip model was tested for type 1 diabetes, which comprised anastomosing micro-capillaries developed from rodent pancreatic islets for insulin secretion. Hence, such organ-on-a-chip scientific leaps can lead the future of 3D-BP in the direction of a human-on-a-chip model [125]. The limitation of using a conventional single-cell type scaffold was resolved by a recent scaffold-free human breast cancer model, bioprinted using the NovoGen Bioprinting TM platform developed by Organovo Company. The model was engineered to perform clinical drug testing of tamoxifen; it had a thorough heterogeneous cellular tissue construct composed of mesenchymal stem cell-differentiated adipose cells, endothelial cells and mammary fibroblasts. The viability model was two weeks in vitro and the chemotherapeutic profile of the cells was investigated through ATP luciferase assay; distinct compartmentalization of the several cellular layers with capillary formation was observed on histomorphological assays [132]. The 3D-BP of tumor models has been successful in studying the immune response and the mechanistic interactions among the surrounding cells in the tumor environment, the action of the novel biologics (antigen and antibody), drugs, and customized T cells with specific signaling molecules for personalized immunotherapy treatment [133]. Meanwhile, futuristic 3D-BP models can fabricate the native patient-derived tumors cells with the engineered T cells to test their efficacy in deteriorating the tumor [134]. However, it is certainly a challenging task to involve insights into the immunogenic mechanism of T cells in the tumor environment, which may lead to new experimental immunotherapies for cancer [135]. As a conclusive remark, integration of 3D-BP with allied technologies such as microarrays, AI, IoT based models, microfluidics and bioprinted organ/human-on-a-chip models would shorten the pipeline of pre-clinical drug testing trials and produce a viable choice of organ substitutes [136,137].

The mainstay of tissue repair is the potency of the seed cells that are used in tissue repair and regenerative medicine. Nevertheless, their usage is associated with certain drawbacks such as decreased cell viability, reduced regeneration potency, and immune histocompatibility issues. Further, they cannot be universally applied for all type of recipients for transplantation and application in regenerative medicines. Adoption of other types of cell-signaling molecules with paracrine effects seems to the better viable option. Exosomes are potential factors that can replicate the functions of the stem cell, and they are critical components involved in immune response, tissue repair and angiogenesis. Moreover, they act as extracellular nanovesicles for the transportation of mRNA, proteins and other biologically active molecules. Due to these functional aspects, they have been adopted in tissue repair and reconstruction. Several preclinical studies have reported the application of exosomes as nano vesicles used in the field of regenerative medicine and tissue engineering. The prospects of exosome-laden scaffolds for preclinical applications in tissue repair have been studied in bone/cartilage repair and reconstruction, skin and vascular tissue regeneration, and organ transplantations [138]. Recent studies have reported that the MSCs discharge exosomes (MSC-exos) that are composed of biological molecules which exhibit potency for tissue repair. Exosome tissue scaffolds are advantageous because they retain a relative number of the the MSC exosomes at the repair site, which improves the functional roles and maintain structural integrity. Further, their presence at the site impacts and regulates morphological modifications in the micro-architecture. Moreover, they are seamlessly absorbed and actively function to stimulate the tissue regeneration process [139]. 

Several clinical studies have been reported based on exosome scaffolds for cardiac regeneration using the exosome parental cells (EPC) of bone marrow-derived endothelial progenitor cells [140], while EPCs of human umbilical cord-derived MSCs have been used for diabetic wound healing [141]. Similarly, EPCs of human adipose-derived stem cells have been successfully employed for bone regeneration [142], while human-induced pluripotent stem cell-derived MSCs have been used for cartilage regeneration [143] and so on. In cases of specific requirements, engineered exosomes such as miR-375 loaded in hydrogels were implanted in a rat skull defective model; the continuous release of exosomes into the target site remarkably improved the bone regeneration [144]. Meanwhile, genetically engineered exosomes loaded with nucleic acid drugs drastically improved tissue repair and optimized the treatment duration in patients [145]. In parallel, drug- and small molecule-loaded exosomes can be continuously released into the target site for improved and enhanced repair of the cartilaginous tissue [146]. Due to the above discussed benefits, drug-loaded exosome-laden scaffolds will be an effective choice for cell-free alternatives in tissue engineering and regenerative therapy in the future.

## 12. Conclusions

3D bioprinting is a technique that works with bioinks made of cells and other biomaterials to mimic natural tissues and construct artificial tissues, scaffold and organs through fabrication methods. This technological intervention has caused tangible progress and insightful improvements in regenerative medicines and tissue engineering. In this review article, the latest benchmarks of several tissues/organs such as skin, bone and cartilage, liver, kidney, smooth muscles, cardiac and neural tissues have been elaborately discussed; their limitations, pipelines and advantages are highlighted. These studies have undoubtedly established a strong foundation for organ constructs and have presented promising goals for in vivo organ replacements in future; however, we also presented the lacunae in the in vitro models which will serve as a research gap for upcoming research to find solutions. 3D bioprinting technologies involve extrusion, inkjet, laser and pressure-based bioprinting; these have become the commonest and most progressive methods in tissue engineering and disease modelling. Further, this review also considers successful attempts at testing drugs, pharmacokinetics and physiological functions in in vitro models. These advancements have now made it possible to produce 3D bioprinted patches, which have great potential for directly repairing infected tissues and replacing surgical procedures, which will substantially alleviate the burden of the lack of organ transplants. Limitations such as vasculature, innervations and micro-mimicking the architecture of the tissue constructs have been overcome through the integration of microfluidics with 3D bioprinting technology. Though the forthcoming ideas of 3D bioprinting are promising, there are many technical encounters to overcome, including optimization studies on bioinks and cell sources before they are widely implemented in clinical practice.

## Figures and Tables

**Table 1:** Clinical trials of 3D-BP tissue constructs in in vivo models.

S. No	Type of Tissue	Clinical Models	Reference
1.	Skin tissue	Primary human dermal keratinocytes were fabricated with dermal equivalents and epidermis-like structures were developed	[62]
2.	Skin tissue	Free-form fabrication (FFF) 3D bioprinting technique was adopted to engineer human plasma-derived bilayered skin using human fibroblasts (hFBs) and keratinocytes (hKCs) retrieved from skin biopsies were implanted in immunodeficient athymic mice	[63]
3.	Skin tissue	3D-BP pigmented human skin constructs were engineered with keratinocytes, melanocytes and fibroblasts obtained from donors, and they exhibited similar constitutive pigmentation as the skin donors	[64]
4.	Neural tissue	Improved spinal cord regeneration, reduced scar and offered elongated nerve fibers in vivo (transection rat SCI model) using porous scaffolds	[65]
5.	Neural tissue	Polyurethane-based NSCs-based 3D-BP constructs using fused deposition showed improved motor function and survival rate in adult zebrafish with induced traumatic brain injury (TBI) models	[66]
6.	Neural tissue	Gellan gum fabricated with oligodendrocyte-like cell scaffolds implanted in the injured tissues showed less inflammation in hemisection rat spinal cord injury (SCI) model	[67]
7.	Bone tissue	3D-BP models influenced bone tissue regeneration (BTR) in calvarial bone defects in animal models	[68]
8.	Bone tissue	Biodegradable 3D printed scaffolds with controlled release of deferoxamine and developed through a layer-by-layer assembly technique improved angiogenesis and osteogenesis and augumented bone development and reconstruction in animal models	[69]
9.	Bone tissue	Nanoclay-based 3D printed scaffolds stimulated vascular ingrowth in ex vivo conditions and produced bone mineral tissue in vivo models	[54]
10.	Cardiac tissue	Epicardial application of human cardiac-derived progenitor cells (hCMPCs) in a 3D-printed gelatin/hyaluronic acid patch significantly improved cardiac function after myocardial infarction in mouse model	[70]
11.	Cardiac tissue	3D printed complex tissue patch tailored with stem cell-laden decellularized extracellular matrix bioinks worked well for cardiac repair in mouse models Improved cell migration to the infected area, improved cardiac function and reduced cardiac hypertrophy and fibrosis.	[71]
12.	Cardiac tissue	3D bioprinted cardiac tissues with recombinant human tropoelastin were assessed in in vivo models. They further stimulated negligible inflammatory response and showed effective biodegradation in vivo in subcutaneously implanted rats	[55]

**Table 2:** Representation of different types of bioinks used in recreating several 3D bioprinted tissue constructs.

Bioinks	Type of Cell Lines	Bioprinted Material	Bioprinter	Reference
Protein-Based Bio-Inks				
Collagen	Encapsulated keratinocytes and fibroblasts	3D skin tissue	Laser-based 3D bioprinters	Koch et al. [84]
Collagen droplets	Smooth muscle cells (SMCs)	Valve-based droplet ejector system	Skin tissue	Moon et al. [85]
Collagen–agarose blend	Mesenchymal stem cells (MSCs)	Extrusion-based	Skin tissue	Duarte Campos et al. [86]
Gelatin-alginate composite	Pre-osteoblasts and Human adipose tissue-derived stem cells (ASCS)	3D tubular bone constructs	3D porous cellular blocks	Yeo et al. [87]
Gelatin-alginate composite	Encapsulate myoblasts	3D tubular bone constructs	Soft tissue constructs	Zhang et al. [88]
Gelatin/alginate bioink with hydroxyapatite (HAp)	Encapsulate myoblasts	3D tubular bone constructs	Syringe tip heaters in extrusion printers	Wüst et al. [89]
Gelatin-alginate composite +	Encapsulated smooth muscle cells (SMCs) in valve root part and valve leaflet interstitial cells	Cell-laden aortic valve conduits	Extrusion-based bioprinter	Duan et al. [90]
Agarose, Alginate, GelMA, and BioINK	Articular cartilage- resident chondroprogenitor cells (ACPCs)	Cartilage tissue constructs	Extrusion-based bioprinting system (3D bioplotter)	Levato et al. [91]
Fibrin and alginate	Encapsulated chondrocytes	Cartilage tissue	Inkjet bioprinter	Nakamura et al. [92]
Fibrinogen	Encapsulated endothelial colony-forming cells (ECFCs) were in a fibrinogen-HA mixture	3D assembly of multi-cellular arrays.	Laser-based 3D bioprinters	Gruene et al. [93]
fibrinogen with PEG or a PEG-geIatin mixture	Human microvascular endothelial cells (HMVEC)	Micro-vasculature networks	Thermal inkjet printing technology	Cui and Boland [94]
Silk fibroin and gelatin	primary chondrocytes from porcine	Cartilage tissue constructs	Micro-extrusion bioprinter	Singh et al. [95]
Hyaluronic acid (hyaluronan, HA)	Human bone marrow-derived mesenchymal stromal cells (MSCs)	Cartilage tissue constructs	Extruder and microvalve-based print	Hauptstein et al. [96]
Hyaluronic acid methacrylate (HAMA)	Pancreatic extracellular matrix (pECM)	3D-printed islet organoid	Extrusion-based bioprinting system	Wang et al. [97]
**dECM-based bioinks**				
Silk-dECM construct + TGF-β encapsulated	Decellularized extracellular matrix (SF-dECM bioinks) mixed with bone marrow mesenchymal stem cells (BMSCs)	Cartilage tissue	Extrusion-based bioprinting system	Zhang et al. [98]
dECM-based bioinks	Decellularized adipose, heart, and cartilage tissue structures	Various tissues	Multi-head tissue-organ building system (MtoBS)	Pati et al. [53]
GelMA with liver dECM.	Encapsulated human-induced hepatocytes (hiHep cells)	Liver tissue	DLP (digital light processing) is a 3D printing technology	Mao et al. [99]
dECM-based bioink	Primary ovarian Cells	3D Primary ovarian cell-laden	Bio-architect 3D bioprinter	Zheng et al. [100]
Ru/SPS with dECM	Human turbinate mesenchymal stromal cells (hTMSCs)	Various tissues	Extrusion-based printing and DLP bioprinting system	Kim et al. [101]
Matrigel with alginate	Vascular endothelial growth factor (VEGF)	Human endothelial progenitor cells (EPCs) laden constricts	Pneumatic dispensing system enabled	Poldervaart et al. [102]
**Synthetic polymer-based bioink**				
Graphene–polyurethane composite	Neural stem cells	Tissue constructs for neural tissue	Conventional bio-printer	Huang et al. [103]
Silicon, ceramic, cellulose, metal, and carbon-based nano materials.	Respective cell-laden hydrogels	Bone and cartilage tissue constructs	Multi-head tissue-organ building system (MtoBS)	Cai et al. [104]
Albumen/Na Alg composite	Human umbilical vein endothelial cells	Organ 3D printing	Extrusion-based 3D bioprinting system	Liu et al. [105]
ECM/AMP hydrogel containing 2% octapeptide FEFEFKFK	Encapsulated dental pulp stem cells (DPSCs)	Cranio-maxillofacial Bone Tissue	Microvalve bioprinting	Dubey et al. [106]
Poly(vinyl alcohol) (PVA)	Nanocomposite PVA/GO-HAp	Artificial cartilage re-constructs	Extrusion-based 3D printing	Meng et al. [107]
Au NPs with thiol-modified hyaluronic acid and gelatin (AuNP-sECMs)	NIH-3T3 cells	Vascular	Extrusion-based printing	Skardal et al. [108]
AgNPs in hydrogels	Chondrocytes	Cyborg organs/cartilage	Extrusion-based printing	Hassan et al. [109]
Magnetic iron oxide nanoparticles	Porcine aortic endothelial cells	Vasculature networks	Hybrid nano- printing system	Yildirim and Arslan-Yildiz [110]
Cryo bioink	Red blood cells	Vasculature	Extrusion-based printing	El Assal et al. [111]
Genetically modified phage	MC3T3-E1	Bones	Extrusion-based printing	Deo et al. [112]

## Data Availability

The data presented in this study are available on request from the corresponding authors.
